# Pediatric spinal glioblastoma of the conus medullaris: a case report of long survival

**DOI:** 10.1186/s40880-016-0107-1

**Published:** 2016-05-09

**Authors:** Antonella Cacchione, Angela Mastronuzzi, Maria Giuseppina Cefalo, Giovanna Stefania Colafati, Francesca Diomedi-Camassei, Michele Rizzi, Alessandro De Benedictis, Andrea Carai

**Affiliations:** Department of Pediatric Hematology-Oncology, Bambino Gesù Children’s Hospital, IRCCS, Piazza Sant’Onofrio 4, 00165 Rome, Italy; Neuroradiology Unit, Bambino Gesù Children’s Hospital, Piazza Sant’Onofrio 4, 00165 Rome, Italy; Division of Pathology, Bambino Gesù Children’s Hospital, Piazza Sant’Onofrio 4, 00165 Rome, Italy; Department of Neurosurgery, Fondazione Istituto Neurologico “Carlo Besta”, IRCCS, Milan, Italy; Neurosurgery Unit, Department of Neuroscience and Neurorehabilitation, Bambino Gesù Children’s Hospital, Piazza Sant’Onofrio 4, 00165 Rome, Italy

**Keywords:** Spinal cord cancer, Glioblastoma multiforme, Children, Multidisciplinary treatment, Prognosis

## Abstract

High-grade gliomas of the spinal cord represent a rare entity in children. Their biology, behavior, and controversial treatment options have been discussed in a few pediatric cases. These tumors are associated with severe disability and poor prognosis. We report a case of a 4-year-old child diagnosed with an isolated glioblastoma multiforme of the conus medullaris. The patient underwent subtotal surgical excision, followed by adjuvant radiotherapy and oral chemotherapy. He is alive with mild neurologic deficits at 52 months after diagnosis. We describe the peculiar characteristics of this rare condition in pediatric oncology. We also provide an overview of current multidisciplinary therapeutic approaches and prognostic factors for this disease.

## Background

Primary spinal cord tumors are rare in children and account for less than 1% of all central nervous system (CNS) cancers [1]. In adults, spinal neoplastic lesions are mostly represented by extramedullary tumors (almost 80% of cases), whereas the rate of intramedullary tumors in children reaches up to 35% of all spinal neoplasms [2].

Low-grade astrocytomas represent the most frequently found histological type of intramedullary lesion (50%–88% of cases), approximately 1%–3% are high-grade gliomas, whereas non-glial tumors are less common [3]. Spinal glioblastoma multiforme (GBM) is a highly malignant CNS tumor that is clinically, histologically, and genetically heterogeneous. Spinal GBM is only rarely seen in children and represents 1%–5% of all GBMs [1].

In this paper, we report a rare case of spinal GBM of the conus medullaris that occurred in a 4-year-old child. We further describe the peculiar characteristics of this rare entity in childhood and illustrate the current multidisciplinary therapeutic approaches.

## Case presentation

A 4-year-old boy reported a 6-month history of night-time worsening pain of both of the lower limbs and a subsequent appearance of urinary retention and weight loss. Neurologic examination demonstrated right leg paresis with bilateral hyporeflexia of the lower limbs. There were no neurologic abnormalities of the upper limbs and cranial nerves. No saddle anesthesia was observed.

**Fig. 1:**
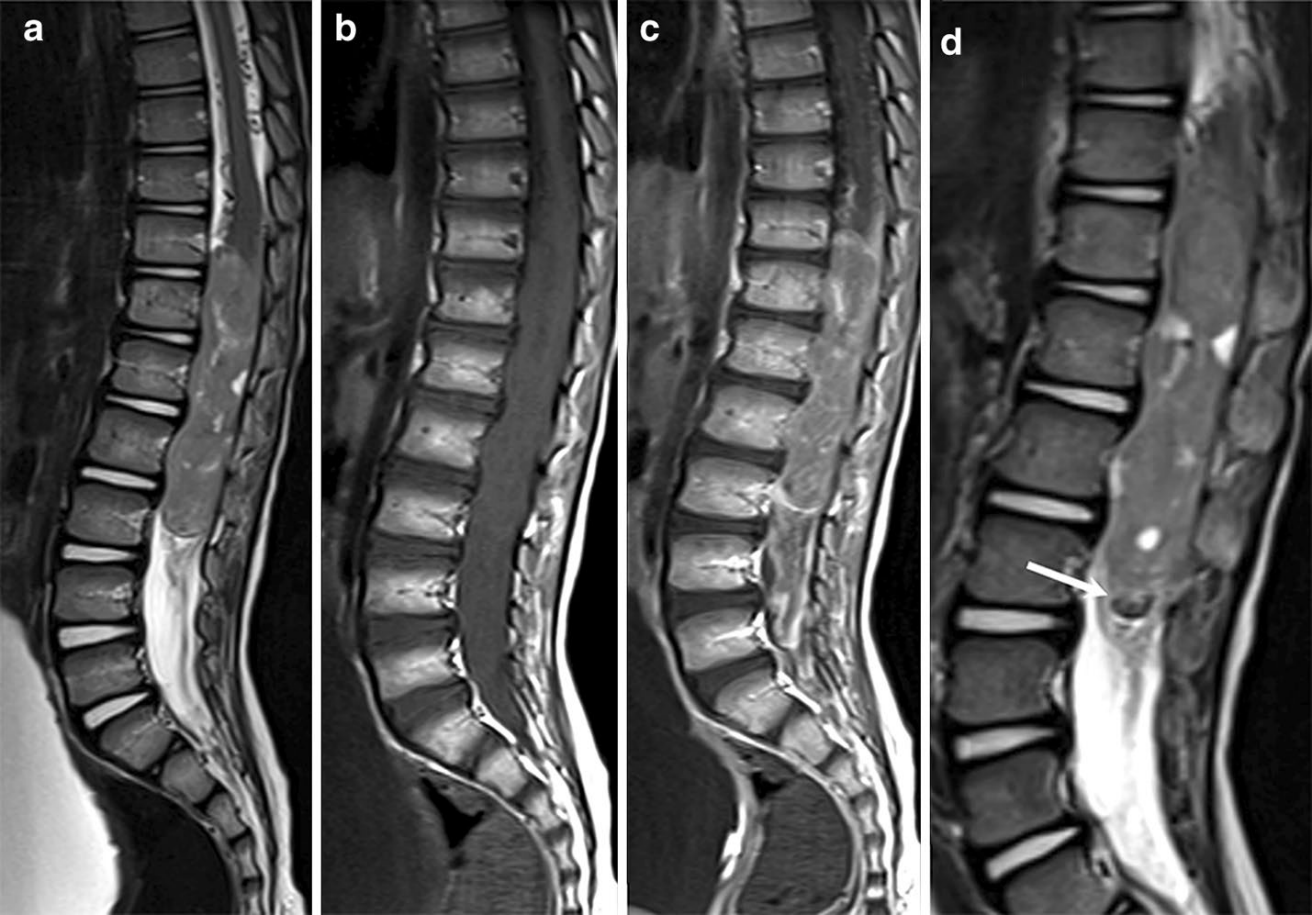
Magnetic resonance imaging (MRI) of a 4-year-old boy with spinal glioblastoma multiforme (GBM) of the conus medullaris at the time of diagnosis. MRI shows a large heterogeneous mass extensively filling the spinal canal between T11 and L3. The lesion shows hyperintense and inhomogeneous signal intensity on Sagittal T2-weighted images (**a**) and isointense signal intensity on Sagittal T1-weighted images (**b**). After gadolinium (Gd) injection, a diffuse, inhomogeneous enhancement of the tumor is observed, and the tecal sac is filled by abundant enhancing tissue enveloping the conus medullaris and cauda equina (**c**). The regions of hypointensity within the tumor and along the inferior margin of the lesion shown on sagittal T2-weighted images suggest tumoral bleeding (*arrow*) (**d**)

Brain and spine magnetic resonance imaging (MRI) revealed a large heterogeneous ovoid mass of 8 cm in the major diameter that filled the spinal canal between T11 and L3 (Fig. 1a, b) with inhomogeneous enhancement of the tumor area (Fig. 1c). A perilesional hemorrhagic area was observed (Fig. 1d). No other lesions were observed by brain or spinal MRI.

**Fig. 2:**
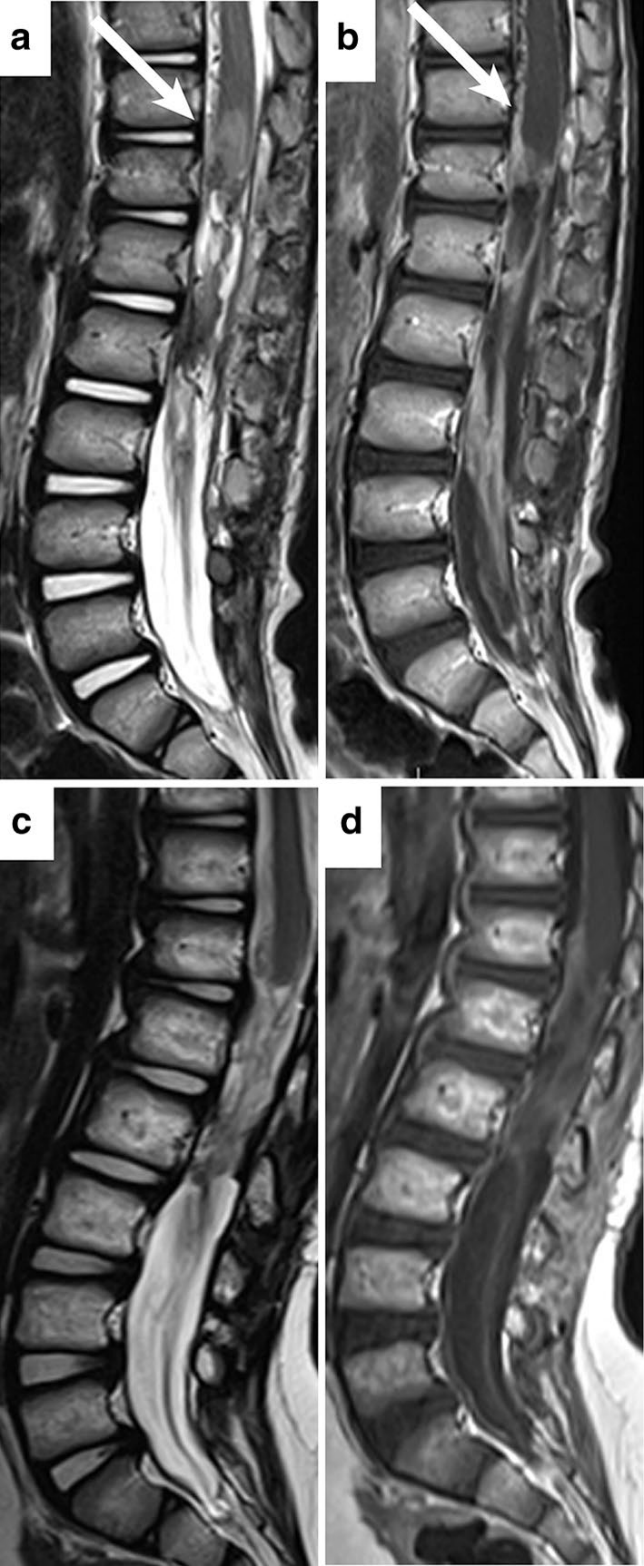
Postoperative MRI of the boy with spinal GBM of the conus medullaris. Postoperative MRI shows a subtotal resection of the primary lesion and leptomeningeal involvement at the level of the conus medullaris and cauda equine (*arrows*) (**a**); after Gd injection, there is no contrast enhancement (*arrows*) (**b**). Two years after surgery, MRI shows the absence of relapses (**c**) and stable persistence of the previously documented enhancement along the right anterolateral conus medullaris (level D10-D12) and along the cauda equina (**d**)

**Fig. 3:**
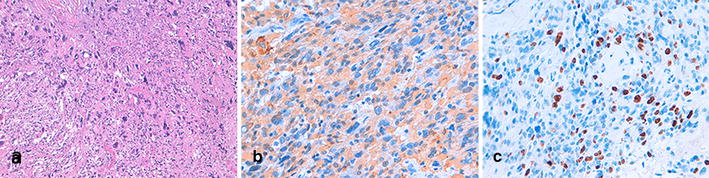
Histopatologic features of the spinal GBM of the conus medullaris. Microscopy demonstrates a neoplastic proliferation of polymorphous glial cells characterized by anisocaryosis and atypical mitosis. **a** Focal necrosis, calcifications, and areas with multinucleated cells are present (×40). **b** Immunohistochemistry shows positivity for glial fibrillary acidic protein (GFAP) and S100 and negativity for sinaptofisine, neurofilaments, and the epithelial membrane antigen (EMA) (×63). **c** The proliferation index (anti-Ki67) is 15% (×63)

Microsurgical subtotal excision was performed with intraoperative neurophysiologic monitoring. After opening the dura, a brownish-red, highly vascularized solid mass appeared. At the end of the procedure, the excision appeared to be subtotal, as confirmed by postoperative MRI (Fig. 2a, b). The patient’s clinical status worsened after surgery, with worse paraparesis on the right side. Hypoesthesia with a D11 level and urinary incontinence were also observed. Pathology was suggestive of GBM (Fig. 3).

Mutational analyses for tumor protein 53 (*TP53*), lysine 27 on histone H3 (*H3K27*), and isocitrate dehydrogenase 1 (*IDH1*) genes were performed, but no overexpression or mutations were identified.

The child underwent focal radiotherapy of the conus medullaris lesion (a dose of 4500 cGy delivered in 25 fractions) with temozolomide (TMZ, with a daily dose of 75 mg/m^2^ during irradiation; a daily dose of 150 mg/m^2^ for 5 days, with a 3-week interval for the first cycle after irradiation; a daily dose of 200 mg/m^2^ for 5 days, repeated every 28 days for the following 11 cycles) [4]. No severe adverse effects related to adjuvant treatment were observed and, in particular, there was no delay in treatment because of hematologic toxicities.

The patient underwent neuro-rehabilitation with a gradual improvement of his motor weakness. He was soon able to stand and walk with a walking device. At 52 months after surgery, his motor impairment was almost completely resolved, although the patient continued to have a mild urinary retention syndrome. Late follow-up imaging confirmed complete remission in this patient (Fig. 2c, d).

## Discussion

Pediatric intramedullary GBM are uncommon clinical entities. The commonly reported locations of GBM are the cervicothoracic segments, with the cervical spine being the most affected region, followed by the thoracic spine [5]. The isolated involvement of the conus medullaris is very infrequent, representing only 3 % of all pediatric cases, whereas holocordal presentations and intracranial dissemination are often described. The tumor typically tends to spread via the subarachnoid space due to the proximity of the neoplastic tissue to the cerebrospinal fluid space. Survival ranges from 4 to 16 months with a median survival of 12 months [6].

Clinical features depend on the region of the spinal cord involved and the growth rate of the tumor, irrespective of the histological subtype. The most common symptoms in children include pain, motor regression, gait abnormalities, torticollis, and progressive kyphoscoliosis.

Based on imaging, the criteria for the differential diagnosis of these lesions in children have been determined [7]. Our case showed MRI features compatible with a high-grade neoplasm, including hemorrhage involving the lower pole, the so-called “cap sign,” the presence of multiple cysts, and leptomeningeal involvement [7].

Similar to the treatment strategy for brain lesions, gross total resection of the spinal cord lesion, confirmed by early postoperative MRI, followed by adjuvant treatment consisting of radiotherapy and chemotherapy, has been recommended [7].

Historically, pediatric GBM has been treated with adjuvant radiotherapy followed by cytotoxic drugs either as single agents or in combination. Unfortunately, none of the chemotherapeutic regimens has been demonstrated to be superior over the others [8].

Due to the lack of alternative treatments with superior clinical efficacy, radiotherapy in combination with concomitant and adjuvant TMZ is widely used by pediatric neuro-oncologists based on the efficacy of this strategy in adults [9]. This treatment regimen is also used in patients with spinal GBM.

Because of the high propensity of spinal GBM to disseminate, whole spine irradiation has been proposed. However, the role of prophylactic craniospinal irradiation remains unclear [10]. Taking the patient’s age and minimal residual disease into consideration, we decided to perform focal irradiation at the 52-month follow-up visit when there was no evidence of dissemination.

Pediatric GBM usually has a grim prognosis. Survival ranges from 6 to 16 months, with a mean survival of 12 months after the diagnosis [1]. An exceptionally long survival of 144 months has been reported for a patient with spinal GBM [11].

The extent and location of the lesion as well as the feasibility of a gross total resection, especially in younger patients, are the factors that most affect a patient’s prognosis [12]. Despite the presence of several positive prognostic factors, survival remains poor [13].

Even after 52 months of follow-up, our patient is not considered to be cured. Intensive rehabilitation aiming at optimal clinical recovery has promoted a good quality of life and the ability to perform daily life activities without support.

The good clinical outcome observed in our case might partially be due to the absence of known negative prognostic molecular markers described in pediatric high-grade gliomas such as TP53 and H3K27 [14, 15]. The identification of new molecular markers helps to define the prognosis of GBM in daily practice [16].
